# The British Journals, &c.: Medical Jurisprudence and Toxicology

**Published:** 1842-04

**Authors:** 


### MEDICAL JURISPRUDENCE AND TOXICOLOGY.

Recovery after 2 drachms of arsenic were taken. A boy, seventeen years of age, took ʒij of arsenic, but made it known within a quarter of an hour afterwards. Six grains of tartar emetic were given; vomiting was kept up for two hours; he was bled copiously; injections were thrown up into the bowels, and a blister was applied to the stomach to relieve pain in that organ. On the fourth day the boy was so well that all apprehensions respecting him were laid aside.—J. Toogood, Esq., *Prov. Med. and Surgical Journal*, No. xiv. *Jan.* 1, 1842.

---

Static Lung Tests. This paper is the sequel of a former one designed to prove the insufficiency of the static lung tests, or rather of the data hitherto employed in discussing the value of these tests. Thus as regards the weight of the lungs of still-born children, Schmitt makes the maximum as high as 1661 grains, Orfila only 5861; while, as regards the minimum, Haartman, in four still-born children, found the smallest weight 1066 grains, while Lecieux states it at 340. If the various tables of the authors just named, along with those of Jörg, Eisenstein and Zebitsch, Devergie, Taylor, the Procès Verbal, and the author of this paper, be collated, it will be seen that in more than one instance, the maxima in still-born children fall but little short of those of children that have lived one month; while, in one case, the highest number in the still-born greatly exceeds the highest in those born alive. The author points out the serious inconveniences to which such discrepancies may lead in medico-legal investigations.

Ploucquet's test, or that which is drawn from the weight of the lungs as compared with that of the rest of the body, has some advantages over the one founded on the absolute weight of the former, inasmuch as the results seem more uniform.

The following are the general conclusions of this valuable paper:

"*Weight of the Lungs.*—1. The weight of the lungs of still-born children of the same age varies within wide limits; the chief causes of difference being the sex and the weight of the body.

"2. The weight of the lungs in mature still-born children is as follows: greatest weight, 1661; least weight, 340; average weight, 874.

"3. The weight of the lungs in mature still-born children of the male and female sex respectively is as follows: greatest weight, 1661, 1492; least weight, 360, 340; average weight, 950, 809.

"4. The weight of the lungs in children who have respired also varies within wide limits; the chief causes of difference, in addition to those which affect still-born children, being the degree and duration of respiration.

"5. In children who have survived their birth one month or less, the highest recorded weight is 2440 grains; the lowest 432 grains; and the average 1072 grains.

"6. The weight of the lungs for males and females respectively, at the same ages, is as follows: greatest weight, 2440, 1745; least weight, 432, 479; average weight, 1121, 982.

"7. The weight of the lungs increases with the increasing perfection of the respiration, but is very slightly augmented by imperfect respiration.

"8. The weight of the lungs also increases with the duration of the respiration, but appears to be less when respiration has continued more than one hour and less than twelve, than when it has lasted less than one hour.

"9. The mean weight of the lungs in mature children who have lived one month or less exceeds the mean weight in mature still-born children by somewhat less than one fourth, the numbers being 574 and 1072.

"10. The average and extreme values drawn from small numbers of facts differ widely from each other, and cannot be depended upon for medico-legal purposes.

"11. The average values cannot be safely employed as standards of comparison, and the extreme values admit of very rare application.

"12. If the absolute weight of the lungs is employed as a test of respiration, the value obtained in an individual case ought to be compared with the average or extreme numbers obtained for the same weight of body.

"The following propositions have an important bearing on Ploucquet's Test:

"1. The weight of the lungs both before and after respiration increases with the weight of the body; but the proportion which the lungs bear to the body decreases as the weight of the body increases

"2. For the same weight of body the weight of the lungs varies within wide limits, and *vice versa*, for the same weight of lungs the weight of the body varies within wide limits. This variation is more considerable after respiration than before it.

"3. The weight of the body in still-born children is greater than in children born alive; the former exceeding the latter by nearly one third.

"4. The weight of the lungs is subject to much greater variation than that of the body.

"5. The weight of the lungs is much greater in the male than in the female.

"1 *Ploucquet's Test.* The proportion which the weight of the lungs bears to that of the body, like the absolute weight of the lungs, varies within wide limits; the proportion in mature still-born children being as follows: greatest proportion, 1 : 24; least proportion, 1 : 176; average proportion, 1 : 57.

"2. The proportion in males and females respectively is as follows: greatest proportion, 1 : 24, 1 : 36; least proportion, 1 : 176, 1 : 119; average proportion, 1 : 53, 1 : 63.

"3. In children who have survived their birth one month or less, the highest recorded proportion is 1 : 19; the lowest, 1 : 132; and the average, 1 : 38.

"4. The proportion for males and females respectively at the same ages is as follows: greatest proportion, 1 : 19, 1 : 19; least proportion, 1 : 132, 1 : 96; average proportion, 1 : 35, 1 : 43.

"5. The proportion which the lungs bear to the body increases with the increasing perfection of the respiration, but is very slightly augmented by imperfect respiration.

" 6. The proportion also increases with the duration of the respiration, but appears to be less when respiration has continued more than one hour and less than twelve, than when it has lasted less than one hour.

" 7. The average proportion in mature children who have lived one month or less, exceeds that in mature still-born children; the numbers being 1 : 57 before respiration, and 1 : 38 after respiration.

" 8. The proportions calculated from a small number of facts differ widely from each other, and cannot be depended upon for medico-legal purposes.

" 9. The average proportions cannot be safely employed as standards of comparison, and the extreme values, though more to be depended on than the highest and lowest weight of the lungs, are of very limited application.

" 10. If the average or extreme proportions are employed as standards of comparison, the proportion obtained in any individual case must be compared with the average or extreme numbers calculated for the same weight of body.

The observations contained in the present essay lend strong confirmation to the unfavorable opinion expressed on a former occasion of the static-lung tests as tests of respiration. Whether employed to distinguish respiration from non-respiration, or respiration from inflation, they are alike insufficient, except in cases of extremely rare occurrence, where we can make use of the extreme values. On the supposition that the question of inflation has no place, the static lung tests are as unnecessary as they are useless; if we have proved that either respiration or inflation has taken place, they can only be employed with advantage in the extremely rare instances just alluded to, viz. where we can employ the extreme values. Hence, then, the proposition which concludes my first essay requires to be slightly modified, and will stand thus.

" The static lung tests are utterly useless for all practical purposes, and ought not to be relied on in medico-legal enquiries except in rare instances, where the extreme values can be employed."—Dr. Guy, *Edin. Med. and Surg. Journal*, No. cl. *Jan.* 1, 1842.

---

Insufficiency of Albumen as an Antidote to Corrosive Sublimate. After pointing out Orfila's mistake in supposing albumen to be an antidote to the bichloride of mercury, the author remarks: " It is known that certain ammoniacal salts, if present in sufficient quantity, *entirely prevent the precipitation of corrosive sublimate by albumen.* Muriate of ammonia is one of these, a salt existing in the animal fluids. Muriate of soda, another salt found universally in animal fluids, has the same effect, though in a less degree. I might add many others; but already enough has been said to show *the inadequacy of albumen as a test for corrosive sublimate.* As regards the detection of mercury, the dismissal of albumen from our list of reagents is not to be regretted, while we possess the means of reproducing that base *in its metallic state*, a condition that ought, as in the case of arsenic, to be insisted on at all times by our public tribunals."—John T. Barry, *Pharmaceut. Journ.*, No. vii. *Jan.* 1, 1842.

---

Case of Poisoning with a Minim and a half of Laudanum. The author delivered a poor woman of a fine female child, and before leaving her, prescribed a draught, containing twelve minims of tincture of opium, in an ounce of pimento water, with a view of relieving the after-pains; this was on Monday, and on the Wednesday following, the nurse gave the child half a teaspoonful of the draught, in order to compose it and keep it from crying. The author found it in a state of profound coma, and notwithstanding all the means put in requisition, the infant died fourteen hours from the taking of the dose. Dr Christison has reported a case of a like fatal result, in consequence of a child, 3 days old, having had the fourth part of a mixture containing ten drops of laudanum—a quantity greater than that of the present case. The author justly infers, from his case, the great caution requisite in administering opium to infants.—Mr. Everest, *Lancet*, No. xxii. *Feb.* 26, 1842.

